# Immunohistochemical Detection of *Propionibacterium acnes* in Granulomas for Differentiating Sarcoidosis from Other Granulomatous Diseases Utilizing an Automated System with a Commercially Available PAB Antibody

**DOI:** 10.3390/microorganisms9081668

**Published:** 2021-08-04

**Authors:** Takuma Isshiki, Sakae Homma, Yoshinobu Eishi, Matsuko Yabe, Kazuya Koyama, Yasuhiko Nishioka, Tetsuo Yamaguchi, Keisuke Uchida, Kurara Yamamoto, Kenichi Ohashi, Atsushi Arakawa, Kazutoshi Shibuya, Susumu Sakamoto, Kazuma Kishi

**Affiliations:** 1 Department of Respiratory Medicine, School of Medicine, Toho University, Tokyo 143-8541, Japan; takuma.isshiki@med.toho-u.ac.jp (T.I.); susumu1029@med.toho-u.ac.jp (S.S.); kazuma.kishi@med.toho-u.ac.jp (K.K.); 2 Department of Advanced and Integrated Interstitial Lung Diseases Research, School of Medicine, Toho University, Tokyo 143-8540, Japan; mam.yabe050712@docomo.ne.jp (M.Y.); kkoyama02028@gmail.com (K.K.); 3 Department of Human Pathology, Graduate School and Faculty of Medicine, Tokyo Medical and Dental University, Tokyo 113-8519, Japan; eishi.yoshi@gmail.com (Y.E.); yamatet@icloud.com (T.Y.); uchipath@tmd.ac.jp (K.U.); kakipth1@tmd.ac.jp (K.Y.); kohashi.pth1@tmd.ac.jp (K.O.); 4 Department of Respiratory Medicine and Rheumatology, Graduate School of Biomedical Sciences, Tokushima University, Tokushima 770-8503, Japan; yasuhiko@tokushima-u.ac.jp; 5 Department of Pulmonology, Shinjuku Tsurukame Clinic, Tokyo 151-0053, Japan; 6 Department of Human Pathology, School of Medicine, Juntendo University, Tokyo 113-8421, Japan; aatsushi@juntendo.ac.jp; 7 Department of Pathology, School of Medicine, Toho University, Tokyo 143-8541, Japan; kaz@med.toho-u.ac.jp

**Keywords:** sarcoidosis, granuloma, pathogenesis, *P. acnes*, *Propionibacterium acnes*, *Cutibacterium acnes*, immunohistochemistry, PAB antibody

## Abstract

*Propionibacterium acnes* is implicated in the pathogenesis of sarcoidosis. We investigated the usefulness of immunohistochemistry (IHC) with a commercially available *P. acnes*-specific monoclonal antibody (PAB antibody) for differentiating sarcoidosis from other granulomatous diseases. Formalin-fixed paraffin-embedded tissue samples from 94 sarcoidosis patients and 30 control patients with other granulomatous diseases were examined by the original manual IHC method. We also compared the detection frequency of *P. acnes* in sarcoid granulomas between manual and automated IHC methods. *P. acnes* was detected in sarcoid granulomas of samples obtained by transbronchial lung biopsy (64%), video-associated thoracic surgery (67%), endobronchial-ultrasound-guided transbronchial-needle aspiration (32%), lymph node biopsy (80%), and skin biopsy (80%) from sarcoidosis patients, but not in any non-sarcoid granulomas of the samples obtained from control patients. *P. acnes* outside granulomas, however, was frequently detected in both groups. The detection status of *P. acnes* in granulomas did not correlate with the clinical characteristics of sarcoidosis patients. The automated Leica system exhibited the best detection sensitivity (72%) and almost an identical localization for *P. acnes* in sarcoid granulomas compared with the manual method. IHC with a PAB antibody is useful for differentiating sarcoidosis from other granulomatous diseases by detecting *P. acnes* in granulomas. An automated method by the Leica system can be used in pathology laboratories for differential diagnosis of granulomas by IHC with the PAB antibody.

## 1. Introduction

Sarcoidosis is a systemic granulomatous disorder characterized by the formation of noncaseating epithelioid cell granulomas at sites of the disease, such as the eyes, skin, lungs, and lymph nodes [1]. Although the precise mechanisms are not fully understood, granuloma formation in sarcoidosis is recognized as a hypersensitive immune response to causative agents, such as infectious microorganisms [2]. Among the potential infectious agents, *Mycobacterium tuberculosis* and *Propionibacterium acnes*—currently referred to as *Cutibacterium acnes* [3]—are frequently reported to be associated with the pathogenesis of sarcoidosis [4]. Potential latent infection by both microorganisms, however, has complicated the findings of molecular and immunologic studies [5].

According to the principle of granuloma formation, that the causative agent is present or has been present in granulomas [6], histologic identification of infectious or non-infectious agents in granulomas is important to link the agents with the pathogenesis of granuloma formation. Although microbial DNA of *M. tuberculosis* [7,8,9] and *P. acnes* [10] has been reported in granulomas, the detection of microbial DNA does not necessarily indicate co-localization of microbial protein antigens in the granulomas. *P. acnes* is so far the only microorganism repeatedly detected in sarcoid granulomas by immunohistochemistry (IHC), apart from one study [11] in which *M. tuberculosis* heat shock proteins were located in sarcoid lesions by IHC. *P. acnes* in granulomas has been found in the tissues of various organs of patients with sarcoidosis, including the lungs [12,13], lymph nodes [14], heart [15], eyes [16,17], and nervous system [18,19]. A *P. acnes*-specific monoclonal (PAB) antibody used for IHC to detect *P. acnes* in granulomas reacts with lipoteichoic acid, the cell membrane constituent of the bacterium, in formalin-fixed and paraffin-embedded (FFPE) tissues [20].

Several reports have suggested the potential usefulness of IHC with a PAB antibody for diagnosing sarcoidosis. The control subjects in past reports, however, were mostly limited to subjects with tuberculosis and sarcoid reaction. Furthermore, the association between the detection status of *P. acnes* in granulomas and the clinical characteristics was not fully analyzed. IHC with the PAB antibody in previous reports was mainly conducted in a single institution and the IHC method was not validated in other institutions.

While the original method for PAB antibody detection is a manual method, a Dutch research group recently reported the results of IHC with a PAB antibody in sarcoid tissues using an automated system from Ventana with modifications [21]. In their study of Dutch patients, *P. acnes* in granulomas was more frequently detected in patients with chronic diseases requiring treatment than in those without, whereas the detection frequency was generally lower than in Japanese and German cohorts [20]. The difference in the detection frequency of *P. acnes* in granulomas may be due to the lower detection sensitivity of the automated IHC method. Thus, both manual and automated methods for IHC with a PAB antibody should be evaluated in a single study.

The main purpose of the present study was to elucidate the usefulness of IHC with a PAB antibody for differentiating sarcoidosis from other various granulomatous diseases. In addition, we examined the detection sensitivity of several automated IHC methods using the Leica and Ventana systems in comparison with the original manual method.

## 2. Materials and Methods

### 2.1. Patients

We obtained FFPE tissue sections of 94 patients with sarcoidosis and of 30 patients with other granulomatous diseases as controls, who were treated at Toho University and Tokushima University between 2006 and 2020. Sarcoidosis was diagnosed according to clinical findings consistent with sarcoidosis and the presence of noncaseating granulomas in biopsy or surgically resected samples [22]. To analyze the association between the clinical course after diagnosis of sarcoidosis and detection status of *P. acnes* in granulomas, we divided the sarcoidosis patients into stable and unstable groups according to the criteria described in an earlier report [21]. The stable group was defined as patients with natural improvement, no worsening, or no requirement for systemic treatment such as corticosteroids. The unstable group was defined as patients with definite worsening of the disease or need for systemic treatment. The diagnosis of other granulomatous diseases was confirmed in each hospital clinically and pathologically by several clinicians before the study. The institutional review board of Toho University Graduate School of Medicine approved this study (M21018_20167_18066). The opt-out method was adopted, and informed consent was waived because this study was retrospective and noninvasive, and because patient anonymity was secured.

### 2.2. Baseline Characteristics of Sarcoidosis and Control Patients

The clinical profiles of the 94 patients with sarcoidosis at diagnosis are shown in Table 1. The diagnosis was histologically confirmed in all patients. Chest X-ray stages mainly comprised stage I and II, and only one patient showed lung fibrosis. Bronchoalveolar lavage fluid findings of a high CD4/8 ratio and lymphocyte count were consistent with the diagnosis of sarcoidosis. Baseline characteristics of 30 control patients with other granulomatous diseases are shown in Table 2. In these control patients, granulomas were detected in at least one site by pathology. Among the patients with a sarcoid reaction, five had lung cancer, one had breast cancer, one had malignant lymphoma, and one had pancreatic cancer.

### 2.3. Immunohistochemistry

The commercially available PAB antibody (D371-3, MBL, Nagoya, Japan) was used for both the manual and automated IHC methods. FFPE tissues were cut 4 μm thick, de-paraffinized, and rehydrated. The original manual IHC method was performed as described previously [20]. The tissue sections were microwaved (Microwave Processor type MI-77; Azumaya Medical Instruments Inc., Tokyo, Japan) in 10 mmol/L citrated buffer (pH6.0) for 40 min at 97 °C and treated with 3% hydrogen peroxide in methanol for 10 min. After treatment with normal blocking serum (Vectastain Universal Elite ABC kit, #PK-7200; Vector Laboratories, Burlingame, CA, USA), the sections were incubated with PAB antibody (1:8000) overnight in a humidified chamber at room temperature. Sections were incubated with biotinylated secondary antibody for 30 min, and then with streptavidin–peroxidase complex (Vectastain Universal Elite ABC kit) for 30 min. Finally, the signal was developed by peroxidase substrate diaminobenzidine (DAB) (Nichirei Bioscience, Tokyo, Japan) and the sections were counterstained by Mayer’s hematoxylin.

Automated IHC by Leica BOND-III (Leica Microsystems Inc., Tokyo, Japan) was performed using a BOND Polymer Refine Detection kit (#DS9800, Leica Microsystems Inc.). Deparaffinization, peroxidase inhibition, antigen retrieval with BOND Epitope Retrieval Solution 1 (#AR9961, Leica Microsystems Inc.) at 100 °C for 60 min, incubation with primary antibody (PAB antibody, 1:500) at room temperature for 8 min, and counterstaining were performed according to the manufacturer’s protocol.

Automated IHC using the VENTANA BenchMark Ultra (Ventana Medical Systems Inc., Tucson, AZ, USA) was performed according to three different methods. “Ventana method A” is a conventional protocol of the Ventana system using the OptiView DAB IHC Detection Kit (#760-700, Ventana Medical Systems Inc.). Deparaffinization, peroxidase inhibition, antigen retrieval with CC1 solution (#950-124, Ventana Medical Systems Inc.) at 100 °C for 64 min, incubation with primary antibody (PAB antibody, 1:4000) at room temperature for 30 min, and counterstaining were all performed according to the manufacturer’s protocol. “Ventana method B” is a modified protocol developed in our laboratory to avoid the use of mineral oil during the reaction with PAB antibody, because we found in our preliminary study that the mineral oil used in the Ventana system inhibits the reaction of the PAB antibody. In the method B protocol, the slides are taken out of the machine just before reaction with the PAB antibody. After washing off the mineral oil with EZ buffer and tap water, the slides were returned to the machine for reaction with the PAB antibody. Other steps were the same as in method A. “Ventana method C” is a modification of method B introduced by the Dutch group [21] to differentiate DAB signals by IHC from background coal pigments in tissue samples. In the method C protocol, an ultraView Universal Alkaline Phosphatase Red Detection Kit (#760-501, Ventana Medical Systems) was used instead of OptiView DAB Detection kit. The other steps were the same as in method B.

Cases with at least one small brown or red round/dot granule within or outside the granulomas were defined as positive according to the localization of positive signal, respectively. Histopathologic assessment was performed by three pathologists (S.H., Y.E., and K.O.). For cases with different evaluations among the three observers, the detection status was determined by discussion among the observers.

### 2.4. Data Analysis

Continuous variables were analyzed with the unpaired t-test when comparing two groups. Categorical variables were compared with the Chi-square test and Fisher exact test. All *p*-values are two-sided, and a *p*-value of less than 0.05 was considered to indicate statistical significance. Statistical analysis was performed using PRISM version 8 (GraphPad Software., San Diego, CA, USA).

## 3. Results

### 3.1. Detection Frequency of P. acnes in Tissues by Location

**Figure 1:**
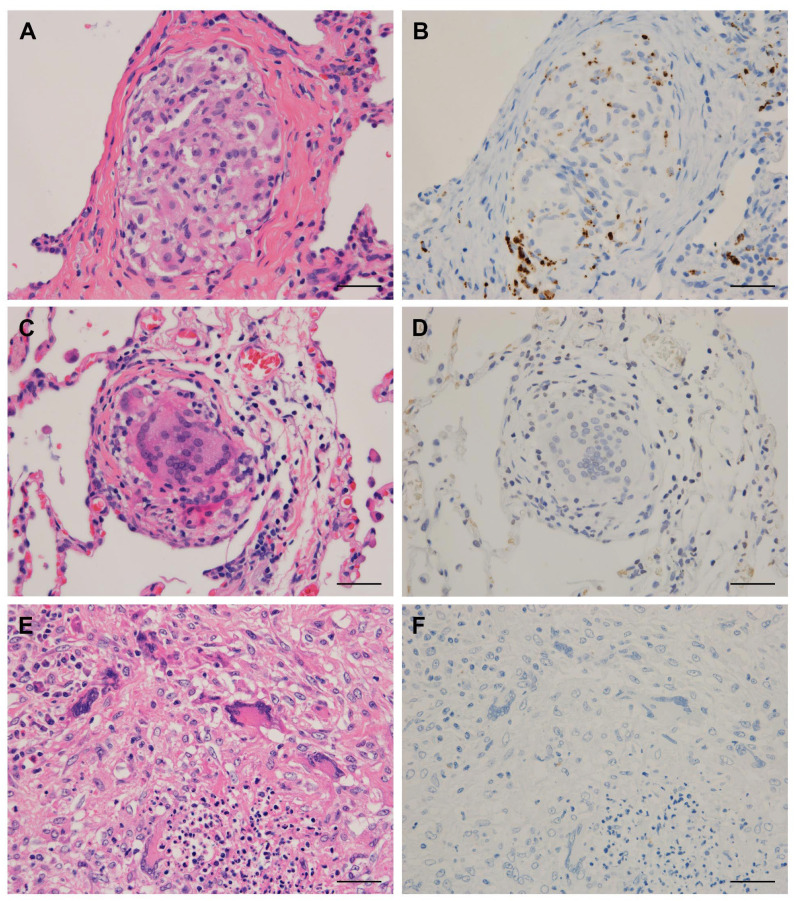
*P. acnes* detected in granulomas of the lungs from patients with sarcoidosis, hypersensitivity pneumonia, or granulomatosis with polyangiitis. Hematoxylin–eosin staining and immunohistochemistry with *P. acnes*-specific monoclonal antibody (PAB) are shown pairwise. PAB-reactive *P. acnes* (resulting in brown color) is observed in a solitary non-caseating epithelioid cell granuloma found in a TBLB sample of sarcoidosis (**A**,**B**). No signal of PAB-reactivity is observed in a small alveolar granuloma found in a VATS sample of hypersensitivity pneumonia (**C**,**D**) and in granulomatous inflammation found in a VATS sample of granulomatosis with polyangiitis (**E**,**F**). Scale bar: 50 µm.

**Figure 2:**
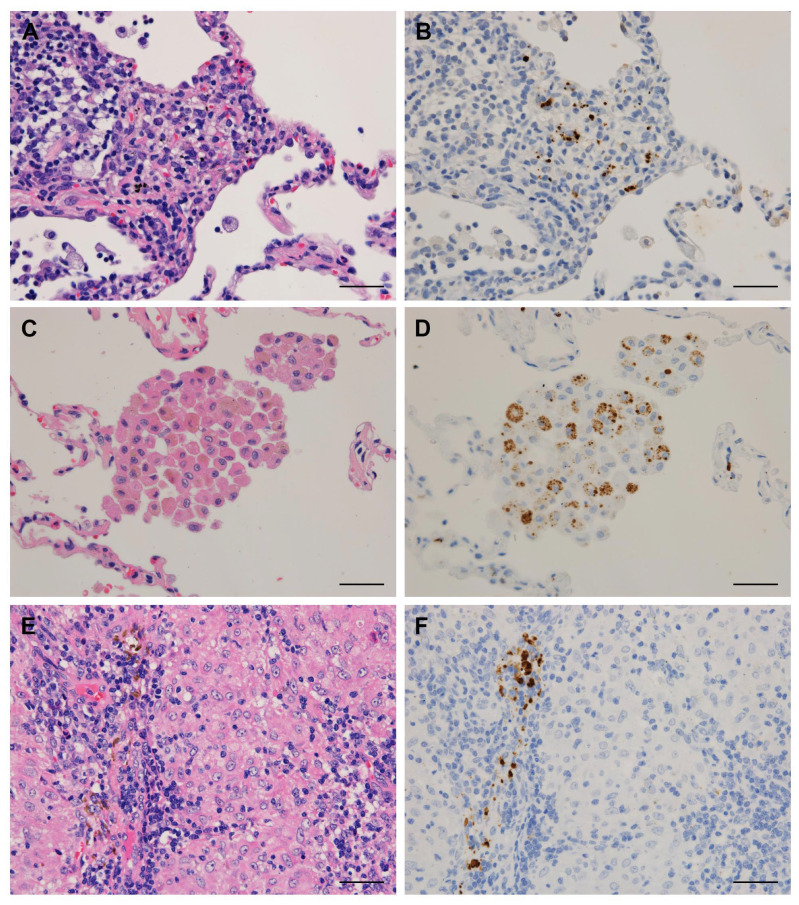
*P. acnes* detected in other areas outside granulomas such as lung interstitial or alveolar macrophages and lymph node sinus macrophages. Hematoxylin–eosin staining and immunohistochemistry with *P. acnes*-specific monoclonal antibody (PAB) are shown pairwise. PAB-reactive *P. acnes* are observed in a cluster of interstitial macrophages with inflammation found in a TBLB sample of hypersensitivity pneumonia (**A**,**B**), and in a ball-like cluster of eosinophilic alveolar macrophages found in a VATS sample of granulomatosis with polyangiitis (**C**,**D**), both of which were histologically differentiated from granulomas. In the lymph nodes with sarcoid reaction (**E**,**F**), PAB-reactive *P. acnes* including large ovoid Hamazaki–Wesenberg bodies are observed in the narrowed lymphatic sinus between granulomas with no *P. acnes* detected inside. Scale bar: 50 µm.

Tissue samples of the lung, lymph node, and skin from sarcoidosis and control patients were obtained by transbronchial lung biopsy (TBLB), video-associated thoracic surgery (VATS), endobronchial-ultrasound-guided transbronchial-needle aspiration, lymph node biopsy or resection, or skin biopsy. The frequency of detection of *P. acnes* in these tissues using the manual IHC method is summarized in Table 3, according to the location of the positive signal. *P. acnes* in granulomas was detected in 64% of TBLB and 67% of VATS lung samples, 32% of EBUS-TBNA and 80% of biopsy lymph node samples, and 80% of skin samples from sarcoidosis patients, but not in any of the samples from control patients with other granulomatous diseases, including hypersensitivity pneumonia (HP) and granulomatosis with polyangiitis (GPA) (Figure 1). The detection frequency of *P. acnes* in sarcoid and non-sarcoid granulomas was significantly different when compared between 42 sarcoidosis and 17 control lung samples (64% vs. 0%, *p* < 0.0001) and between 36 sarcoidosis and 10 control lymph node samples (39% vs. 0%, *p* = 0.020). On the other hand, *P. acnes* was frequently detected in other areas outside the granulomas such as interstitial or alveolar macrophages and lymphatic sinus macrophages in both sarcoidosis and control samples (Figure 2), with no significant difference in the detection frequency at either location (Table 3).

### 3.2. Clinical Data and Detection Status of P. acnes in Granulomas

Evaluation of the association of the clinical data at diagnosis with the detection status of *P. acnes* in granulomas of tissues from sarcoidosis patients revealed no significant association with serum biomarkers, pulmonary function, or bronchoalveolar lavage fluid findings between groups with or without *P. acnes* detected in granulomas (data not shown).

**Figure 3:**
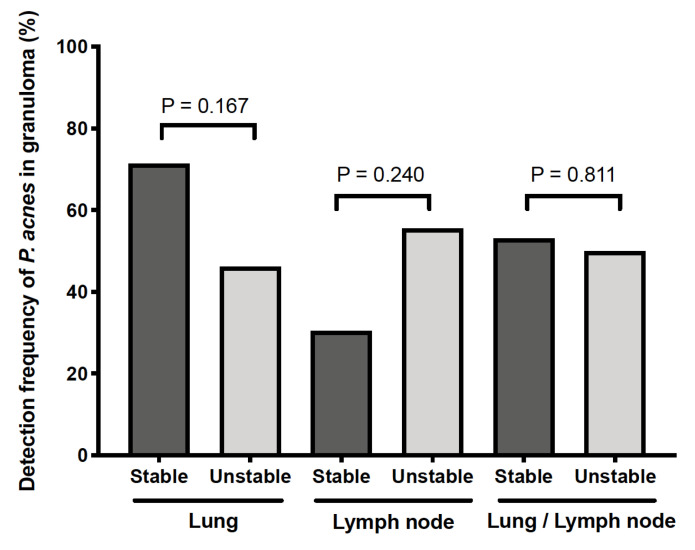
No association between the clinical outcome of sarcoidosis and the detection status of *P. acnes* in granulomas. Sarcoidosis patients were divided into stable and unstable groups (*n* = 49 and 22, respectively) as described in the Materials and Methods. Detection frequency (%) of *P. acnes* in granulomas was compared between the two groups of patients according to the organ used for the evaluation by immunohistochemistry: the lung (*n* = 41), lymph node (*n* = 32), and either lung or lymph node (*n* = 71). No significant difference was observed between the stable and unstable groups, regardless of the organ used for the evaluation of *P. acnes* in granulomas.

As described in the Materials and Methods, we divided sarcoidosis patients into stable and unstable groups according to their clinical course after diagnosis. Patients diagnosed from skin samples (*n* = 18) and patients whose clinical course was untraceable (*n* = 5) were excluded from this analysis. The remaining 71 patients (*n* = 49 for the stable group and *n* = 22 for the unstable group) were included in the analysis. Between the two groups, we compared the detection frequency of *P. acnes* in granulomas of the lungs (*n* = 41), lymph nodes (*n* = 32), or either organ (*n* = 71), respectively; no significant difference in the detection frequency was observed between the stable and unstable groups, regardless of the organ used for evaluation by IHC (Figure 3).

### 3.3. Detection Sensitivity of P. acnes in Granulomas by Automated IHC Methods

**Figure 4:**
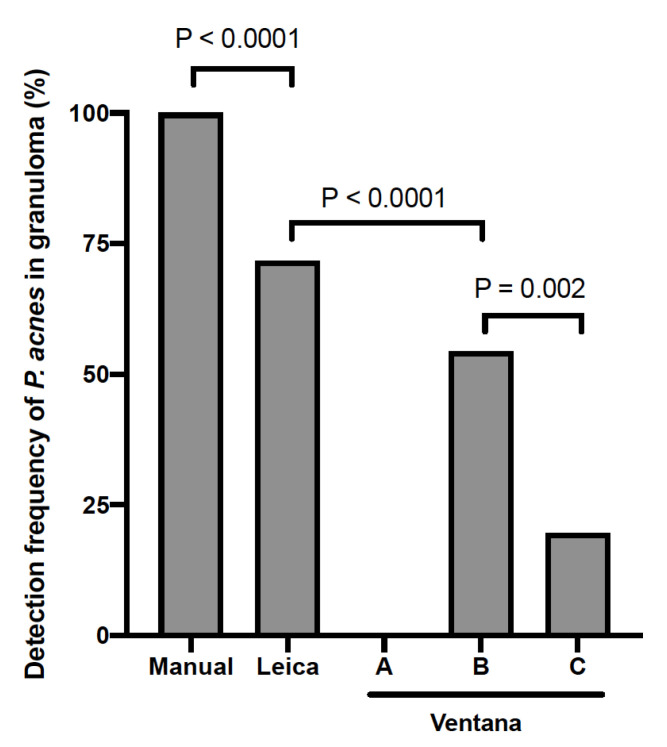
Detection frequency of *P. acnes* in sarcoid granulomas by immunohistochemistry using the Leica and Ventana systems. A total of 46 sarcoid tissue samples with *P. acnes* detected in granulomas by the manual method were used for comparison. Manual: a standard manual method according to the original protocol. Leica: a standard method using Leica Bond-III, Ventana A: a standard method using Ventana BenchMark Ultra, Ventana B: a modified method in which the mineral oil was removed before the primary antibody reaction, and Ventana C: a further modified method in which the detection kit was replaced with an alkaline phosphatase red detection kit.

**Figure 5:**
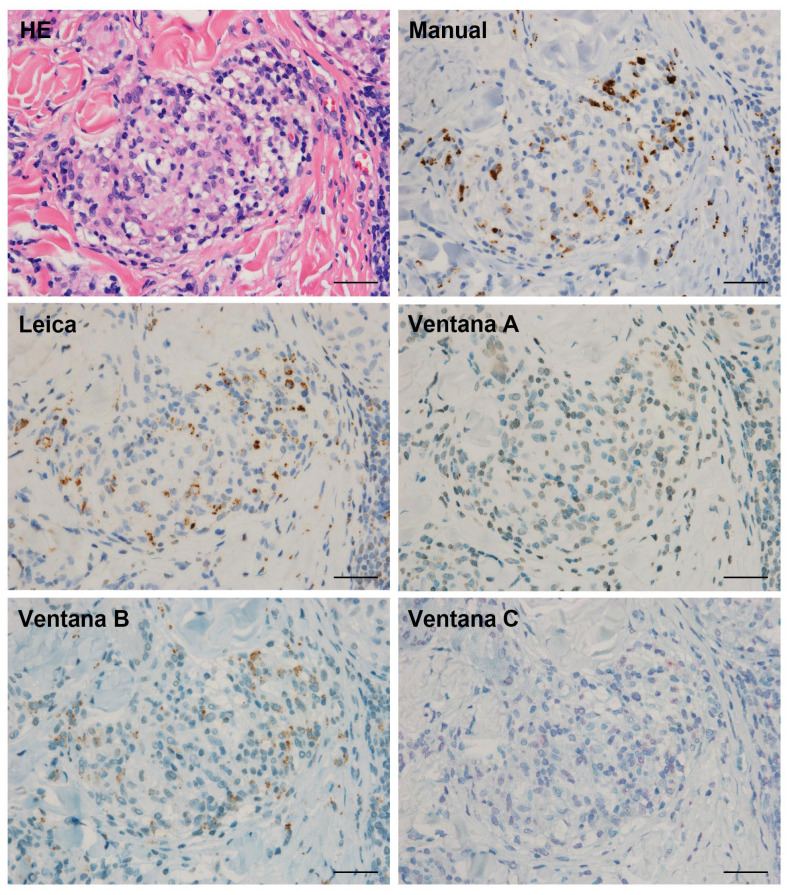
Representative detection status of *P. acnes* in sarcoid granulomas by immunohistochemistry using the manual and automated methods. The results from a skin biopsy sample with many *P. acnes* detected in sarcoid granulomas are presented. HE: Hematoxylin–eosin staining, Manual, Leica, Ventana A, B, C: as described in Figure 4. Almost identical localization of *P. acnes*-specific monoclonal antibody reactivity was observed between the manual method and the automated method using the Leica system, although the intensity of the positive signals in the automated system was generally lower than that in the manual method. Scale bar: 50 µm.

Finally, 46 sarcoid tissue samples with P. acnes detected in granulomas by manual IHC were then also subjected to automated IHC with the PAB antibody using the Leica and Ventana systems. The number of tissue samples with P. acnes detected in granulomas by each automated method is shown according to the tissue origin in Table 4, and the detection sensitivity of the manual method (total number) was compared with that of the two automated methods (Figure 4). Generally, an almost identical localization of PAB-positive signal was obtained by the Leica system with 72% detection sensitivity. No positive signal was detected by the Ventana system (method A). By removing the mineral oil during the reaction with the PAB antibody (method B), reactivity was recovered with 54% detection sensitivity. By changing the detection kit in the modified Ventana system (method C), the detection sensitivity decreased to 20%. In the Ventana system with modifications, the detection sensitivity was higher in method B than in method C (*p* = 0.002). The detection sensitivity of the Leica system was significantly higher than that of the Ventana method B (*p* < 0.0001). Representative IHC results are shown in Figure 5.

## 4. Discussion

The present study demonstrated the usefulness of IHC with a commercially available PAB antibody for differentiating sarcoidosis from other granulomatous diseases by detecting *P. acnes* in granulomas, even when this commensal bacterium is detected in other tissues outside of the granulomas, regardless of the disease status. *P. acnes* was detected in many sarcoid granulomas, but not in any non-sarcoid granulomas, such as observed in tuberculosis, sarcoid reaction, HP, and GPA. These findings support the hypothesis that sarcoid granulomas can be caused by *P. acnes* commensal to the lungs and lymph nodes in sarcoidosis patients with Th1 hypersensitivity to the bacterium [23].

PAB antibody is a *P. acnes*-specific antibody showing no cross-reactivity with other bacteria including mycobacteria, confirmed by Western blot and IHC [20]. In the original study [20], using IHC with a PAB antibody, *P. acnes* in granulomas was detected in 48% TBLB and 74% VATS lung, and 88% lymph node samples from sarcoidosis patients, but not in any of the samples from patients with tuberculosis or a sarcoid reaction. In this study, *P. acnes* in granulomas was detected in 64% TBLB and 67% VATS lung and 80% lymph node samples from sarcoidosis patients by IHC with the commercially available PAB antibody following the original manual protocol with antigen retrieval by microwave. Thus, the results were reproducible between the different institutes and researchers.

On the other hand, the results of *P. acnes* in sarcoid granulomas detected by an automated IHC method (Ventana) with a PAB antibody were recently reported by a group of Dutch researchers [21]; the detection frequency of *P. acnes* in granulomas was generally lower in their Dutch cohort (17%) than in Japanese cohorts. Ethnicity is unlikely to be a cause of the difference because *P. acnes* in sarcoid granulomas was detected in 89% lymph node samples from German patients in the original study by Negi et al. [20]. Thus, we suspected that the difference between the results of the Dutch and Japanese patients was due to a lower sensitivity of the automated IHC method (Ventana method C) used for the study with Dutch patients.

We examined two automated IHC systems from Leica and Ventana, which are used worldwide for diagnostic pathology. The detection sensitivity of *P. acnes* in sarcoid granulomas was generally lower when using the automated IHC methods than when using the manual method; 72% in the Leica system, 54% in Ventana method B, 20% in Ventana method C, and 0% in Ventana method A. The generally lower sensitivities in the automated IHC methods may be due to the different antigen retrieval method (hot plate heating) compared with the original method (microwave). To use the PAB antibody in the Ventana system, the manufacturer’s protocol had to be modified because mineral oil covering the tissue slides was found to inhibit the reaction of the PAB antibody in Ventana method A. Finally, we suggest that the Leica system can be used in pathology laboratories for a differential diagnosis of granulomas by IHC with a PAB antibody, while the manual IHC method is best in terms of detection sensitivity. In the future, we may need to design an exchange program of samples to test the results obtained by the experts’ teams.

*P. acnes* in granulomas was not detected in some tissue samples from sarcoidosis patients. Indeed, even in the identical lesion in which *P. acnes* was detected in granulomas, some granulomas were positive and other granulomas were negative by IHC with the PAB antibody. The heterogenous reactivity of the PAB antibody in sarcoid granulomas may be a cause of the lower detection frequency (32%) in EBUS-TBNA samples with a few granulomas included. In addition, degradation of *P. acnes* by granuloma cells might be related to the heterogenous PAB reactivity in sarcoid granulomas. Negi et al. [20] reported that positive *P. acnes* signals were observed more frequently in immature granulomas compared with mature granulomas, suggesting that *P. acnes* as a cause of the granuloma formation may be degraded and abolished during maturation of the granuloma. These observations suggest that sarcoidosis should be suspected when *P. acnes* is detected in granulomas, whereas sarcoidosis cannot be ruled out when *P. acnes* is not detected in granulomas.

The result of IHC with a PAB antibody for diagnosing sarcoidosis is not a matter of detecting *P. acnes* in tissues, but rather identifying the bacterium in the histologically-proven epithelioid cell granulomas. Accordingly, histologic evaluation of *P. acnes* in granulomas should be carefully performed by pathologists because this commensal bacterium is frequently found in the area outside the granuloma in both sarcoidosis and control groups. *P. acnes* is the most common commensal microorganism in the lungs and lymph nodes of subjects without sarcoidosis [24]. *P. acnes* survives intracellularly and persists in macrophages without intracellular replication [25]. Endogenous reactivation of latent *P. acnes* may occur not only in sarcoidosis patients, but also in patients without Th1 hypersensitivity to the bacterium [26]. Thus, the presence of *P. acnes* in areas other than granulomas does not link the commensal bacterium to the pathogenesis of sarcoidosis. 

In the present study, *P. acnes* was found in interstitial and alveolar macrophages in 88% and 35% lung tissues, respectively, from patients with other granulomatous diseases, including HP and GPA, although no *P. acnes* was detected in these non-sarcoid granulomas. Recently, Beijer et al. [27] reported that *P. acnes* was found in the lung tissue of 57% HP patients and 33% GPA patients and observed inside granulomas of 26% HP patients and 11% GPA patients, concluding that *P. acnes* may be involved in the disease pathogenesis of those granulomatous diseases in a mitogenic way. The difference in the detection frequency of *P. acnes* in HP or GPA granulomas, however, may be caused by different histologic evaluation of granuloma. Indeed, in the present study, a cluster of interstitial or alveolar macrophages with detection of *P. acnes* was occasionally observed and could be differentiated by pathologists from a granuloma. Thus, further studies are needed with a similar defined method used in an international survey to be designed.

Among sarcoidosis patients, the detection status of *P. acnes* in granulomas was not associated with the clinical data at diagnosis. The detection frequency of *P. acnes* in granulomas did not differ in the present study between the unstable and stable sarcoidosis patients with or without the need for treatment. Among the Dutch sarcoidosis patients in the study by Beijer et al. [21], however, there was a significantly higher detection frequency of *P. acnes* in granulomas of patients with chronic disease requiring treatment. Although the reason for the difference in the results between Japanese and Dutch patients remains unknown, the difference may be associated with the lower sensitivity of the automated method (Ventana method C) they used than the manual method we used. Because *P. acnes* is a potential treatment target of sarcoidosis or a biomarker for treatment response, further investigation is needed to address the association between the clinical outcome of diseases and the detection status of *P. acnes* in granulomas using a common IHC method with sufficiently high sensitivity.

## 5. Conclusions

The present study indicated that histologic detection of *P. acnes* in granulomas by IHC with a PAB antibody is useful for differentiating sarcoidosis from other granulomatous diseases. The detection sensitivity of *P. acnes* in granulomas by automated IHC methods is generally lower than that of the original manual method. Automated IHC with a PAB antibody using the Leica system might be applicable, however, for clinical settings.

## Figures and Tables

**Table 1:** Clinical profiles of sarcoidosis patients at diagnosis.

	Sarcoidosis
Number of patients	94
Age, years	52 ± 14
Sex, male, *n* (%)	55 (59%)
Smoking status (current/former/never), *n*	25/36/33
Laboratory data	
ACE, U/L	20.9 ± 7.9
Lysozyme, μg/mL	11.1 ± 4.8
sIL-2R, U/mL	1036 ± 792
Pulmonary function testing	
%FVC, %	107 ± 16
FEV1%, %	81 ± 12
Chest XP stage (0/I/II/III/IV)	1/41/36/15/1
BALF findings	
CD4/8	6.2 ± 5.0
Lymphocyte, %	33 ± 23

Data are presented as mean ± SD. ACE: angiotensin-converting enzyme, sIL-2R: soluble interleukin 2 receptor, FVC, forced vital capacity; FEV1: forced expiratory volume in 1 s; BALF: bronchoalveolar lavage fluid.

**Table 2:** Baseline characteristics of control patients with other granulomatous diseases.

	Control
Number of patients	30
Age, years	62 ± 15
Sex, male, *n* (%)	15 (50%)
Smoking status (current/former/never), *n*	5/10/15
Diseases, *n*	
Tuberculosis	9
Hypersensitivity pneumonia	9
Sarcoid reaction with cancer	8
Granulomatosis with polyangiitis	4

Data are presented as mean ± SD.

**Table 3:** Detection frequency of *P. acnes* in tissues by localization in sarcoidosis patients and control patients with other granulomatous diseases.

Tissue	in Granulomas		in Other Area outside Granulomas					
			Interstitial Macrophages		Alveolar Macrophages		Sinus Macrophages	
	Sarcoidosis	Control	Sarcoidosis	Control	Sarcoidosis	Control	Sarcoidosis	Control
Lung	27/42 (64)	0/17 (0)	36/42 (86)	14/17 (82)	18/42 (43)	6/17 (35)	-	-
TBLB	25/39 (64)	0/6 (0)	34/39 (87)	5/6 (83)	17/39 (44)	2/6 (33)	-	-
VATS	2/3 (67)	0/11 (0)	2/3 (67)	9/11 (82)	1/3 (33)	4/11 (37)	-	-
Lymph node	14/36 (39)	0/10 (0)	-	-	-	-	7/36 (19)	5/10 (50)
EBUS-TBNA	10/31 (32)	0/5 (0)	-	-	-	-	3/31 (10)	1/5 (20)
Biopsy or resection	4/5 (80)	0/5 (0)	-	-	-	-	4/5 (80)	4/5 (80)
Skin	16/20 (80)	0/1 (0)	17/20 (85)	0/1 (0)	-	-	-	-

Data are presented as number (%) of tissue samples with *P. acnes* detected in each location. TBLB: transbronchial lung biopsy, VATS: surgical lung biopsy by video-associated thoracic surgery, EBUS-TBNA: endobronchial-ultrasound-guided transbronchial-needle aspiration.

**Table 4:** Number of tissue samples with *P. acnes* detected in sarcoid granulomas by manual and automated immunohistochemistry methods.

Tissue (Sampling Method)	Manual	Leica	Ventana		
			A	B	C
Lung (TBLB)	18	10	0	9	5
Lung (VATS)	2	2	0	1	0
Lymph node (EBUS-TBNA)	6	5	0	3	0
Lymph node (biopsy)	4	3	0	2	1
Skin (biopsy)	16	13	0	10	3

Data are presented as number of tissue samples with *P. acnes* detected in sarcoid granulomas of each tissue. Manual: a standard manual method according to the original protocol. Leica: a standard method using Leica Bond-III, Ventana A: a standard method using Ventana BenchMark Ultra, Ventana B: a modified method that removed the mineral oil before the primary antibody reaction, and Ventana C: a further modified method that replaced the detection kit with an alkaline phosphatase red detection kit. TBLB, VATS, EBUS-TBNA: as described in Table 3.
